# The Magic Grasp: Motor Expertise in Deception

**DOI:** 10.1371/journal.pone.0016568

**Published:** 2011-02-09

**Authors:** Cristiana Cavina-Pratesi, Gustav Kuhn, Magdalena Ietswaart, A. David Milner

**Affiliations:** 1 Department of Psychology, Durham University, Durham, United Kingdom; 2 Department of Psychology, Brunel University, Middlesex, United Kingdom; 3 Department of Psychology, Northumbria University, Newcastle upon Tyne, United Kingdom; The University of Western Ontario, Canada

## Abstract

**Background:**

Most of us are poor at faking actions. Kinematic studies have shown that when pretending to pick up imagined objects (pantomimed actions), we move and shape our hands quite differently from when grasping real ones. These differences between real and pantomimed actions have been linked to separate brain pathways specialized for different kinds of visuomotor guidance. Yet professional magicians regularly use pantomimed actions to deceive audiences.

**Methodology and Principal Findings:**

In this study, we tested whether, despite their skill, magicians might still show kinematic differences between grasping actions made toward real versus imagined objects. We found that their pantomimed actions in fact closely resembled real grasps when the object was visible (but displaced) (Experiment 1), but failed to do so when the object was absent (Experiment 2).

**Conclusions and Significance:**

We suggest that although the occipito-parietal visuomotor system in the dorsal stream is designed to guide goal-directed actions, prolonged practice may enable it to calibrate actions based on visual inputs displaced from the action.

## Introduction

An actor fighting in a movie scene, a boxer trying to enter the adversary's defences, and a conjurer performing a magic trick, all have in common the ability to deceive other people using body movements. Although deception has been previously studied from the point of view of the person being deceived [1], [2], much less is known about the skills of successful deceivers.

When using “sleight-of-hand”, magicians have to perform simulated actions that are near-indistinguishable from real ones [3]. For example, in a classic “French drop”, a coin gets concealed in one hand instead of being transferred to the other hand. This is one of many sleights of hand in which simulated rather than real grasping actions are performed. Previous studies have shown that although very similar at first glance, such “pantomimed” reach-to-grasp movements in untrained subjects normally differ distinctively from real ones. Characteristically, they are made with a reduced reach velocity and greater movement amplitude during the approach phase, with the handgrip opening to a smaller extent. Such differences have been interpreted by hypothesizing that real and pantomimed actions are governed by different visual brain pathways [4]. Recent neuroimaging data [5] have confirmed this idea, revealing activations within well-documented left parietal “dorsal-stream” areas during the grasping of real objects, but in quite different brain areas during the pantomimed grasping of imaginary objects. This difference is believed to reflect the specialized role of the dorsal stream in goal-directed visuomotor control [6].

**Figure 1:**
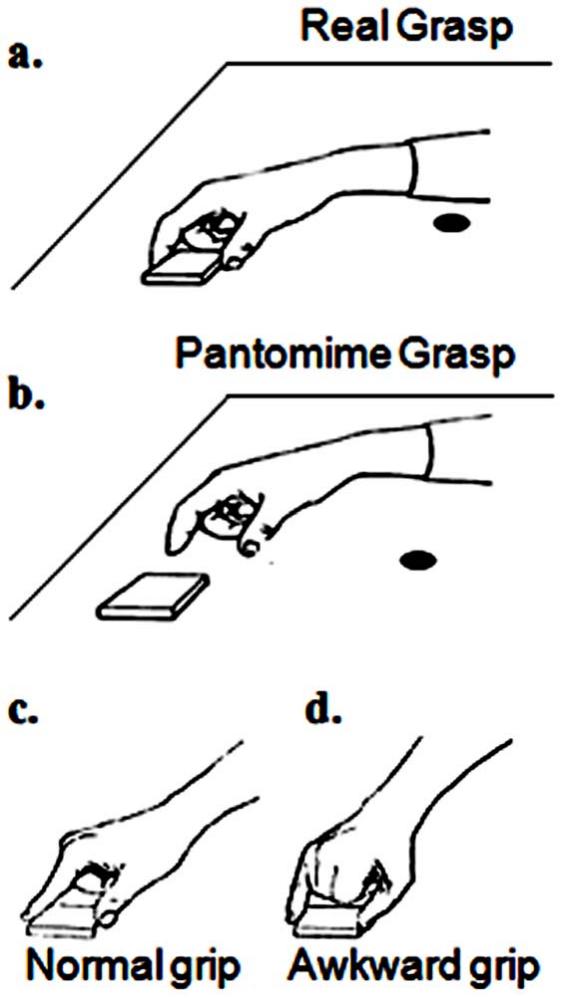
Schematic representations of real (a) and pantomimed (b) reach-to-grasp actions using a normal grip (c) and an awkward grip (d). In Experiment 1 the pantomimed actions were made at a location 10 cm from the object. The black dot represents the starting position.

To date, very little is known about how individuals with extensive experience and practice in deception, such as magicians, execute simulated actions. First of all, is it possible for magicians to perform pantomime actions that are kinematically indistinguishable from real actions? If so, what is the mechanism that allows them to perform such deceptive behaviour? In the present study, we asked whether the normal kinematic differences between real and pantomimed actions would still be apparent in professional magicians, by tracking the positions of their fingers over time as they (and controls) performed the two kinds of actions (Figure 1).

## Materials and Methods

### Experiment 1

Ten professional magicians and 10 control subjects (male, 5 right-handed and 5 left-handed) participated. The two groups were matched for age (t_(9)_ = 0.386, p = 0.7) and Edinburgh laterality index (t_(9)_ = 0.681, p = 0.5) [7]. The ethics committee of Durham University, Department of Psychology approved the experiments described here, and written informed consent was obtained prior to the study in accordance with the principles of the Declaration of Helsinki.

A rectangular block was presented on each trial. Participants were asked either to pick up the object *(real grasp)*, or to pretend to pick up an equivalent object located to one side of it *(pantomimed grasp),* with their dominant hand (Figure 1). The actions were performed either by using a normal precision grip (forefinger and thumb) or an awkward precision grip (little finger and thumb). The awkward grip was introduced to control for grip familiarity [8]. Although magicians are familiar with pantomimed actions using normal grips, magicians and controls were equally unfamiliar with awkward grips.

The objects were placed 30 cm away from the dominant hand, which was held with the fingers in pinched position pressing a start button placed along the body midline. On all trials, the object was positioned 10 cm leftward (right-handed subjects) or rightward (left-handed subjects) from the subject's body midline. On pantomime trials, subjects were asked to imagine that the object was located in a central position, identical for both right and left-handers. Two objects with an identical surface area were used (“small”: 5.0×5.0 cm, and “large”: 8.3×3.0 cm). The experimental session included four blocks of trials: one for each combination of action (real, pantomime) and grip type (normal, awkward). Within each block of 30 trials, the object (large, small) was varied pseudo-randomly.

Liquid Crystal shutter glasses (*Plato System*, Translucent Technologies Inc.) were used to control viewing time. At the beginning of each trial the glasses opened, and after 2 s an auditory start signal instructed participants to pick up the object and place it at the mirror position on the opposite side of the work space. This procedure was used for both real and pantomimed actions. As soon as participants released the start button the glasses closed, preventing visual feedback from the hand. Visual feedback was removed in order to mimic the visual conditions typically experienced by the magicians while performing. Magicians usually avoid looking at the location of the trick as they tend to use their eye gaze to manipulate the audience's attention away from the critical location [9], [10]. Movements were recorded by sampling the position of three markers (thumb, index finger and little finger) at a frequency of 86 Hz, using an electromagnetic tracking system (*Minibird*, Ascension Technology Ltd).

Analyses were performed on reaction time (RT), and on traditional components of the transport phase (movement time: MT, peak velocity: PV, and time to peak velocity: TPV) and grip phase (maximum grip aperture: MGA, time to maximum grip aperture: TMGA) of reach-to-grasp actions.

RT was measured as the time interval between the start signal and movement onset (defined as the time at which the velocity of the thumb marker rose above 50 mm/s). MT was measured as the time between movement onset and movement offset, defined as the time when the velocity of the thumb marker fell below 50 mm/s. PV was defined as the maximum resultant velocity of the thumb marker. MGA was computed as the maximum distance in 3D space between thumb and index (normal grip) or thumb and little finger (awkward grip) markers during the hand movement. TMGA was computed as the time interval between movement onset and MGA. We computed two additional variables, one for the transport component (straightness ratio) and one for the grip component (grip overshoot). Straightness ratio (SR) was calculated as the distance travelled by the hand during the movement divided by the Euclidean distance between the first and the last data point of the reach. “Grip overshoot” was computed as the difference between MGA and the contact grip aperture, which was measured as the distance between the finger markers while holding (or pretending to hold) the object at the end of the reach. Data points just before object lifting (i.e. before speed of the thumb marker rose again above 50 mm/s after landing at the object and before lifting it) were chosen as the clearest contact grip aperture landmark for both the real and the pantomimed grasps.

Data were analyzed using a mixed repeated-measures ANOVA where ACTION (real, pantomimed), GRIP (normal, awkward), and SIZE (large, small) were used as within-subjects factors. GROUP (magicians, controls) was used as a between-subjects factor.

### Experiment 2

Seven magicians and seven controls (males, 5 right-handed and 2 left-handed) participated. The groups did not differ in age (t_(6)_ = 0.166, p = 0.9) or laterality index (t_(6)_ = 0.333, p = 0.8). Four of the magicians and four controls had already participated in Experiment 1. There was no difference in age or laterality index between Experiments 1 and 2 for either the magicians (age: t_(15)_ = 1.23, p = 0.24; LI: t_(15)_ = −0.706, p = 0.5) or the controls (age: t_(15)_ = −1.058, p = 0.2; LI: t_(15)_ = 0.009, p = 0.9). Importantly, the groups of magicians participating in Experiments 1 and 2 were comparable in terms of years of experience (t_(15)_ = 1.90, p = 0.08), number of shows performed per year (t_(15)_ = 1.08, p = 0.3), and average hours of practice per week (t_(15)_ = −0.066, p = 0.95). The ethics committee of Durham University, Department of Psychology approved the experiments described here, and written informed consent was obtained prior to the study in accordance with the principles of the Declaration of Helsinki. Procedures were similar to Experiment 1 except for the following: (i) Real and pantomimed actions were performed using normal grip only; (ii) pantomimed grasping was performed in the absence of the real object; and (iii) the objects were familiar items of well-known size: cell batteries types AA, C, and D. In experiment 1 wooden blocks were used to avoid the confound of object meaning with some objects being more familiar to magicians as compared to normal controls. However, in experiment 2, where object meaning was essential in order to reliably recruit visual imagery of the different objects, real meaningful objects (batteries) were used. Batteries were chosen instead of coins, because the latter are more difficult to pick up due to their thinness, and also because coins are often used by magicians in their work.

The objects measured 5×1.3 cm, 5×2 cm, and 5.7×3.3 cm, respectively, and were placed with the long axis along the fronto-parallel plane. Since the subjects were asked to grasp the objects front-to-back, the AA battery was the “small” (thinnest) object and the D battery the “large” (thickest) one.

For real grasping, objects were labeled with their names (AA, C or D) located below them. For pantomimed grasping, the label was presented alone and participants were asked to imagine the corresponding object as located at the plane of action. The experimental session consisted of 3 fixed-sequence blocks: real, pantomime and mixed (real and pantomime trials intermingled). Real and pantomime blocks contained 21 trials (7 for each stimulus), and the mixed block contained 30 trials (5 for each size and action type). In each block, trials were presented pseudo-randomly.

Data were analyzed using a mixed repeated-measures ANOVA where ACTION (real, pantomimed), BLOCK (fixed, mixed), and SIZE (large, medium, small) were used as within-subjects factors. GROUP (magicians, controls) was used as a between-subjects factor.

## Results

### Experiment 1: results and discussion

**Figure 2:**
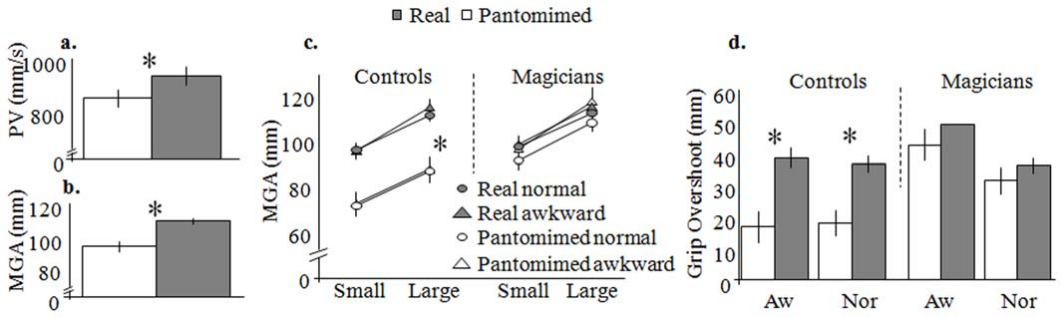
Results of Experiment 1. There was a significantly lower peak velocity (PV) (a) and smaller maximum grip aperture (MGA) (b) for pantomimed than for real grasping movements. c. MGA for real and pantomimed actions executed using normal and awkward grips reported as a function of object size, separately for magicians and controls. While for both groups MGA was larger for the large object than for the small one overall (F_(1.18)_ = 281.60, p = 0.0001), MGA did not significantly differ between real and pantomimed actions for the magicians. d. Grip overshoot for real and pantomimed actions executed with normal and awkward grips for the two groups. While grip overshoot was significantly different for real and pantomimed actions (regardless of grip type) in controls, it failed to reach significance in magicians, who overshoot similarly for both actions. Asterisks highlight significant differences.

Confirming previous results, we found that pantomimed actions had a lower PV (F_(1,18)_ = 74.30, p = 0.0001) and a smaller MGA (F_(1,18)_ = 21.17, p = 0.0001) than real actions (Figure 2a,b). Critically, there was a significant interaction of ACTION x GROUP (F_(1,18)_ = 6.08, p = 0.024), in that MGA did not differ between real and pantomimed actions in the magicians (Figure 2c), for either kind of grasp (normal grip: t_(9)_ = 1.95, p = 0.10; awkward grip: t_(9)_ = -1.53, p = 0.16). However, the control group showed a significantly smaller MGA for pantomimed actions than real actions for both grasp types (normal grip: t_(9)_ = 5.2, p = 0.001; awkward grip: t_(9)_ = -3.8, p = 0.004). There was also a significant ACTION x GROUP interaction for grip overshoot (F_(1,18)_ = 9.49, p = 0.006), see Figure 2d. The extent of overshoot was similar for real and pantomime actions in magicians (normal grip: t_(9)_ = 1.63, p = 0.14; awkward grip: t_(9)_ = 0.45, p = 0.66), but significantly different in controls (with real overshot higher than pantomimed overshoot), again for both grips (normal grip: t_(9)_ = 4.26, p = 0.001; awkward grip: t_(9)_ = 4.63, p = 0.002).

The reaching trajectories showed greater straightness for pantomime grasps than for real ones (F_(1,18)_ = 44.5, p = 0.0001) in that the reaching path for the former was quite direct, whereas the reaching path for the latter bent either leftwards or rightwards depending on handedness. However, and more importantly, there were no GROUP (F_(1,18)_ = 0.245, p = 0.7) or ACTION x GROUP (F_(1,18)_ = 0.61, p = 0.8) effects, suggesting that the directness with which the two groups approached the objects from the starting position was similar, for both real and pantomimed actions.

When asked, the magicians denied applying any intentional strategy. MGA remained constant between the first and second halves of the experimental session (Sessions x Tasks x Groups: F_(1,18)_ = 0.29, p = 0.867), implying that their behaviour was not learned during the experiment.

Given that recent studies have shown a left hemisphere advantage for the visual control of actions [9], [10], and given that we had an equal number of left-handed and right-handed participants (n = 5 each) we checked whether there were any differences in the way the two hands performed the different types of grasps. We ran mixed repeated-measures ANOVAs on both MGA and grip overshoot using ACTION (real, pantomimed), GRIP (normal, awkward), and SIZE (large, small) as within-subjects factors. GROUP (magicians, controls) and HAND (left and right) were used as between-subjects factors. The main factor of HAND failed to reach significance for both measures (MGA: F_(1,16)_ = 0.091, p = 0.8; grip overshoot: F_(1,16)_ = 0.092, p = 0.8) and did not interact with any of the other factors. Although these data speak against a difference between the two grasping hands, no conclusion can be drawn about hemispheric differences as we did not compare the performance of the dominant versus the non dominant hand.

None of the other variables we collected resulted in an ACTION x GROUP interaction (RT: F_(1,18)_ = 0.044, p = 0.5; MT: F_(1,18)_ = 1.4, p = 0.25; PV: F_(1,18)_ = 1.7, p = 0.2, TPV: F_(1,18)_ = 0.139, p = 0.714; TMGA: F_(1,18)_ = 0.045, p = 0.5), suggesting that the success of magicians in pantomiming (1) was restricted to the spatial aspects of hand shaping and (2) was not associated with temporal aspects such as the time to initiate (RT), and to execute either the reach (MT) or the grasping (TMGA) movements.

The results show that, unlike controls, the magicians shaped their hands similarly for real and pantomimed actions. In a prototypical grasp, the fingers open comfortably to exceed the object size before closing around it [11], ensuring a safety margin for grasping the object securely. When the object is absent, as in our pantomime condition, there is no need to adopt this strategy and indeed controls opened their fingers less and only to an extent matching the size of the imagined object, without overshooting. Conversely, magicians showed grip overshoot when grasping the imagined object, just as when approaching the real one. Moreover, the fact that magicians shaped their hands in real-grasping fashion even when pantomiming awkward grasps, suggests that they were applying a generalized skill. It is important to note that the ability of the magicians to pantomime was restricted to the grip component of the reach-to-grasp action. Indeed, the transport phase of the movement was performed less rapidly for the pantomime than for the real tasks. On the basis of the present data it is not possible to tell whether the transport component of the movement was less well simulated because our task instructions focused more on the grip component, or just because to deceive their audience magicians have learned that the movements of the fingers are more important than the movements of the arm. It could also be argued that the transport component is more automatic and less affected by task demands than is the grip component [12]. Regardless of the specific explanation, our data concur with the existence of separate visuomotor streams for shaping the hand and transporting the arm within the parietal cortex [13], [14].

Presumably, with extensive practice in sleight of hand, pantomimed actions in magicians improve to the point where they become indistinguishable from the real thing. One possibility is that this change might be mediated by an improved ability to imagine the objects as being located where the simulated grasp is to be made, thereby providing the visuomotor system with a “quasi-real” goal object. Alternatively, magicians might have somehow learned to recalibrate the visuomotor system itself, so that a normal action can be driven directly by an object in the “wrong” location. To test between these hypotheses, we performed a second experiment, in which magicians and controls performed pantomimed grasping in the complete absence of a real object, requiring them to rely solely on internal visual representations.

It is worth emphasizing that pantomimed grasps performed towards an imagined object by ordinary participants when the real object is either displaced (our Experiment 1) or absent (Experiment 2 below) produce kinematic results that are similar to each other and very different from a real grasp directed toward an object [4]. As previously suggested by Goodale and co-workers [4], in both types of pantomimed action participants rely on an internal representation of the object produced in the ventral stream that is generated quite separately from the visual processing that guides real actions in the dorsal stream. There is a potentially critical difference between the two forms of pantomiming for magicians, however, in that only in the object-present version would they be able to guide their grasps using a putatively recalibrated dorsal stream that allows an action in one location to be driven by an object in another.

### Experiment 2: results and discussion

**Figure 3:**
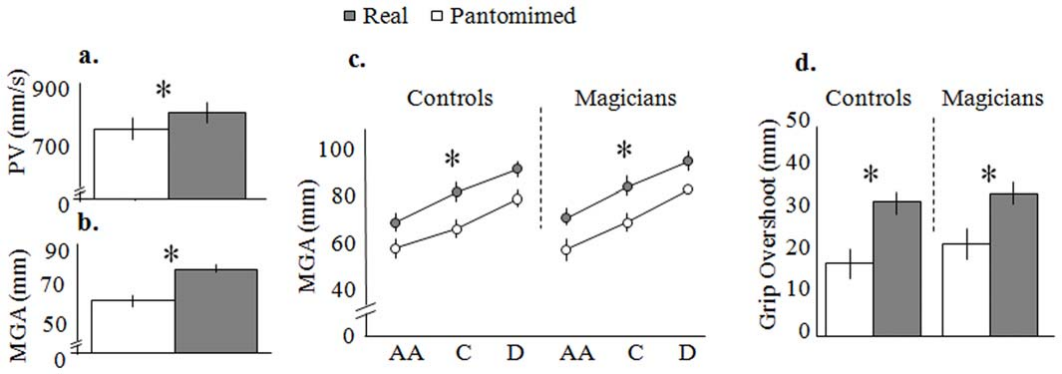
Results of Experiment 2. There was a significantly lower PV (a) and smaller MGA (b) for pantomime than for real grasping movements. c. MGA for real and pantomimed actions in magicians and controls. In both groups, MGA progressively increased with object size (F_(1,13)_ = 225.7, p = 0.0001) and was always larger for real than for pantomimed grasping actions. d. Grip overshoot for real and pantomimed actions is depicted for magicians and controls, showing that it is larger for real actions than for pantomimed ones in both groups. Asterisks highlight significant differences.

Strikingly, pantomimed actions toward an absent object resulted in a lower PV (F_(1,13)_ = 5.51, p = 0.035) and a smaller MGA (F_(1,13)_ = 13.40, p = 0.003) in the magicians, just as in the controls (ACTION x GROUP: F_(1,13)_ = 0.006, p = 0.938): see Figure 3(a,b,c). The interaction was also absent for grip overshoot (F_(1,13)_ = 0.105, p = 0.751), with both magicians and controls overshooting significantly less when pantomiming (Figure 3d). Similarly to Experiment 1, the straightness ratio of the reaching trajectories did not exhibit significant effects either for GROUP (F_(1,14)_ = 0.001, p = 0.98) nor for the interaction ACTION x GROUP (F_(1,14)_ = 1.46, p = 0.25).

As for Experiment 1, none of the other variables collected resulted in an ACTION x GROUP interaction (RT: F_(1,14)_ = 0.383, p = 0.55; MT: F_(1,14)_ = 2.5, p = 1.5; PV: F_(1,14)_ = 0.43, p = 0.53, TPV: F_(1,14)_ = 1.6, p = 0.23; TMGA: F_(1,14)_ = 0.143, p = 0.7).

The results of Experiment 2 show that magicians are no better than controls in pantomimed grasping because of an improved ability to imagine the object as being located where the simulated grasp is to be made. Despite their success in Experiment 1, when they were asked to rely on an internal visual image of the object to be grasped, they failed to perform a pantomimed action that was identical to a real hand movement.

## Discussion

We found that the pantomimed actions of magicians closely resembled their real grasps when the object was visible (but displaced, Experiment 1), but failed to do so when the object was absent (Experiment 2). The results of Experiment 2 suggest that magicians do not benefit from a better capability of imagining the objects to be grasped in the pantomimed task. Instead, we hypothesize that in making realistic pantomime grasps in Experiment 1, the magicians may have used the visual input from the nearby real object to directly drive their visuomotor system. Our proposal would imply that these deception experts do not develop ad-hoc strategies or alternative visuomotor pathways to implement fake actions. We suggest instead that pantomimed grasping actions in magicians are indistinguishable from real ones because the same underlying visuomotor transformations are programmed using the information from the same real objects. To put it in a different way, the pantomimed actions made by magicians may be indistinguishable from real ones because they have learnt that the best way to fake an action is by performing it “for real”. The talent of magicians therefore lies in their ability to use visual input from real objects to calibrate a grasping action toward a separate spatial location (that of the imagined object). Notice that this ability is not related to a different way of transporting the hand. In particular, although it could be argued that magicians might have adjusted their reaching trajectory to get closer to the real object for a better match between object size and grip aperture, our reaching trajectory analyses showed otherwise.

If our interpretation is correct, the next question is, how does this ability develop? We hypothesize that sustained practice in faking actions may render a magician's occipito-parietal visuomotor system more flexible in its use of visual information to drive actions. That is, instead of relying entirely on information coming from the goal object, the system may become able to use information coming from a spatially separate object to calibrate actions. This flexibility might exploit mechanisms similar to those underlying people's ability to adapt to spatially displacing prisms through repeated target-directed movements [15], or like those that underlie the expansion of visual receptive fields during skilled tool use [16]. Such putative plasticity within the visuomotor system itself (as opposed to purely visual or motor plasticity) has seldom been investigated, but may offer a fruitful line of research in the future. Although the neural mechanisms underlying such changes are presently unclear, imaging studies suggest that structural brain modifications take place alongside existing visuomotor areas following the acquisition of new visuomotor skills. For example, clear grey and white matter changes have been identified following extensive juggling practice [17] that are adjacent to brain areas associated with similar but more everyday visuomotor behaviours such as visually guided reaching [14].

It remains an open question as to how the particular motor expertise of magicians is implemented in their brains. One possibility is that it can somehow “piggy-back” onto the pre-existing neural substrates that underlie everyday goal-directed motor expertise. Future research using neuroimaging techniques and virtual lesions using transcranial magnetic stimulation may cast some light on whether or not this does happen.
